# Superoxide dismutase SodB is a protective antigen against *Campylobacter jejuni* colonisation in chickens

**DOI:** 10.1016/j.vaccine.2015.09.100

**Published:** 2015-11-17

**Authors:** Cosmin Chintoan-Uta, Robin L. Cassady-Cain, Halah Al-Haideri, Eleanor Watson, David J. Kelly, David G.E. Smith, Nick H.C. Sparks, Pete Kaiser, Mark P. Stevens

**Affiliations:** a The Roslin Institute and Royal (Dick) School of Veterinary Studies, University of Edinburgh, Easter Bush, Midlothian EH25 9RG, UK; b Department of Molecular Biology and Biotechnology, The University of Sheffield, Sheffield S10 2TN, South Yorkshire, UK; c The Moredun Research Institute, Pentlands Science Park, Penicuik, Midlothian EH26 0PZ, UK; d Institute of Infection, Immunity and Inflammation, College of Medical, Veterinary and Life Sciences, University of Glasgow, Glasgow G12 8TA, UK; e Animal & Veterinary Sciences Group, Scotland's Rural College, Easter Bush, Midlothian EH25 9RG, UK

**Keywords:** *Campylobacter jejuni*, Chickens, Vaccine, Superoxide dismutase, Antibody, Protection

## Abstract

*Campylobacter* is the leading cause of foodborne diarrhoeal illness in the developed world and consumption or handling of contaminated poultry meat is the principal source of infection. Strategies to control *Campylobacter* in broilers prior to slaughter are urgently required and are predicted to limit the incidence of human campylobacteriosis. Towards this aim, a purified recombinant subunit vaccine based on the superoxide dismutase (SodB) protein of *C. jejuni* M1 was developed and tested in White Leghorn birds. Birds were vaccinated on the day of hatch and 14 days later with SodB fused to glutathione *S*-transferase (GST) or purified GST alone. Birds were challenged with *C. jejuni* M1 at 28 days of age and caecal *Campylobacter* counts determined at weekly intervals. Across three independent trials, the vaccine induced a statistically significant 1 log_10_ reduction in caecal *Campylobacter* numbers in vaccinated birds compared to age-matched GST-vaccinated controls. Significant induction of antigen-specific serum IgY was detected in all vaccinated birds, however the magnitude and timing of SodB-specific IgY did not correlate with lower numbers of *C. jejuni*. Antibodies from SodB-vaccinated chickens detected the protein in the periplasm and not membrane fractions or on the bacterial surface, suggesting that the protection observed may not be strictly antibody-mediated. SodB may be useful as a constituent of vaccines for control of *C. jejuni* infection in broiler birds, however modest protection was observed late relative to the life of broiler birds and further studies are required to potentiate the magnitude and timing of protection.

## Highlights

• We tested SodB in chickens as an anti-*Campylobacter* recombinant subunit vaccine.
• It induced a statistically significant reduction in caecal *C. jejuni*.
• Antigen-specific humoral responses did not correlate with protection.
• SodB was not detected on the bacterial surface.
• Protection may not be strictly antibody-dependent.

## 1 Introduction

*Campylobacter* is the leading cause of foodborne diarrhoeal illness in the developed world. In the United Kingdom in 2013 there were 66,575 laboratory-confirmed cases of human campylobacteriosis [1], however for every case captured by national surveillance a further 9.3 are estimated to be undiagnosed in the community and the true incidence may therefore exceed 685,000 cases per annum [2]. The European Food Standards Agency estimated that there are nine million cases of human campylobacteriosis per year across EU27 countries, with the disease and its sequelae (including inflammatory neuropathies and reactive arthritis) causing 0.35 million disability-adjusted life years at a cost of €2.4 billion per annum [3]. Epidemiology unequivocally implicates poultry as the key source of *Campylobacter* affecting humans. Over 90% of laboratory-confirmed human campylobacteriosis is due to *C. jejuni* and source attribution studies indicate that up to 80% of such cases may be due to raw poultry meat [3]. The strategic case to control *Campylobacter* in farmed poultry is compelling, with a year-long UK-wide survey reporting contamination of 73% of raw chicken on retail sale [4]. Such levels are scarcely different from a UK-wide survey in 2007/8 [5]. With a recent census indicating that c. 900 million broilers are reared each year in the UK (c. 60 billion worldwide) the scale of the problem is vast. Though chilling and topical application of chlorinated water, steam, organic acids or bacteriophages can achieve modest reductions in surface contamination, control of *Campylobacter* in broilers prior to slaughter would substantially reduce cross-contamination in the abattoir and pathogen entry into the food chain.

Control of *Campylobacter* may also improve poultry welfare and productivity as recent research indicates that *C. jejuni* elicits prolonged inflammatory responses, damage to intestinal mucosa and diarrhoea in some commercial broiler lines [6]. Moreover, it was reported that *C. jejuni* adversely affects body mass gain in broilers [7] and *Campylobacter*-positive birds are also more likely to exhibit digital dermatitis and signs of colibacillosis [8], though causal links have yet to be formally proven.

Previous studies indicate that various classes of recombinant *Campylobacter* antigens can elicit protection against colonisation in chickens, including major flagellar subunits [9], [10], membrane transport proteins [11], [12] and adhesins [13]. However protection often required large quantities of antigen or was observed too late post-immunisation to be relevant to modern broiler production, where birds often enter the food chain at 6–7 weeks of age. One possible target for improved vaccines is the superoxide dismutase protein SodB. SodB influences intestinal colonisation of chickens by *C. jejuni* [14], and a *sodB* mutant was reported to be defective in entry and survival in cultured intestinal cells [15]. Moreover, a vaccine against *Helicobacter pylori* based on recombinant SodB was protective in a murine model [16]. SodB has a high level of sequence conservation amongst sequenced *Campylobacters* (99%) unlike some candidate antigens evaluated to date. Based on these data, we chose to evaluate a SodB-based subunit vaccine in chickens.

## 2 Materials and methods

### 2.1 Bacterial strains and culture methods

*Escherichia coli* XL1 (Stratagene, USA) was used for maintenance of plasmid constructs and *E. coli* Rosetta BL21 pLysE (Merck Millipore, UK) was used for protein expression. *E. coli* strains were grown in Luria Bertani (LB) broth or agar at 37 °C, unless otherwise indicated, with shaking at 200 rpm for liquid cultures. *C. jejuni* M1 was used as a source of DNA for gene cloning and as the challenge strain in vaccination experiments as described [12]. *C. jejuni* 11168H was used to assess the cross-reactivity and subcellular localisation of SodB. *C. jejuni* strains were grown on modified charcoal-cephoperazone-deoxycholate agar (mCCDA) (Oxoid, UK) or in Mueller-Hinton Broth (MH; Oxoid), at 37 °C in a microaerophilic workstation (Don Whitley Scientific, UK) in a low oxygen atmosphere (5% O_2_, 5% CO_2_ and 90% N_2_). Liquid cultures of *Campylobacter* were grown with shaking at 400 rpm using a table top shaker (IKA, Germany) under low oxygen conditions as above. Antibiotics were used at the final concentrations of 100 μg/ml ampicillin and 34 μg/ml chloramphenicol where appropriate.

### 2.2 Constructs for expression of recombinant antigens

The *C. jejuni* M1 *sodB* gene was amplified using primers 5′ CGCGCGGGATCCATGTTTGAATTAAGAAAATT 3′ (forward) and 5′ CGCGCGGCGGCCGCTTATTTTACAGGGTGAAGTT 3′ (reverse). The *cjaA* gene was amplified using the primers: 5′ GGGCTGGCAAGCCACGTTTGGTG 3′ (forward) and 5′ CCGGGAGCTGCATGTGTCAGAGG 3′ (reverse). Both genes were separately cloned as in-frame C-terminal fusions to glutathione *S*-transferase (GST) in the pGEX-4T1 plasmid (GE Healthcare, UK), through ligation-dependent cloning using the *Bam*HI (5′ end) and *Not*I (3′ end) restriction sites and transformed into *E. coli* XL1 Blue. The *sodB* and *cjaA* genes were re-amplified using the same forward primers described above, but with the reverse primers: 5′ CGCGCGCGCGGTCGACTTATTTTACAGGGTGAAGTT 3′ for *sodB*, and 5′ CGCGCGCGCGGTCGACTTAGATCTTGCCGCCCTCAATA 3′ for *cjaA* and cloned via the *Bam*HI (3′ end) and the *Sal*I (5′ end) restriction sites of the pMal-p2X plasmid (New England Biolabs, UK), to create in-frame fusions with the maltose-binding protein (MBP). The Phusion proof-reading DNA polymerase (Life Sciences, UK) was used to generate amplicons for cloning, using the 2-step cycling conditions as recommended by the manufacturer. All plasmid constructs were verified by dideoxy chain-termination sequencing (Source Bioscience, UK), and transformed into electrocompetent *E. coli* BL21 pLysE Rosetta for protein expression and production.

### 2.3 Expression, purification and validation of recombinant *Campylobacter* antigens

Cultures of 500 ml to 2 l of the *E. coli* BL21 pLysE Rosetta cells encoding GST- and MBP-antigen fusions were inoculated at a 1:100 dilution from a stationary phase overnight culture and incubated for 3 h, with shaking at 200 rpm, at either 28 °C (GST-CjaA and MBP-CjaA) or 37 °C (GST, GST–SodB and MBP–SodB). Cultures were induced with either 0.1 mM isopropyl β-d-1-thiogalactopyranoside (IPTG; Thermo Scientific, UK; GST-CjaA, MBP-CjaA) or 1 mM IPTG (GST, GST–SodB, MBP–SodB) based on pilot studies to optimise expression and solubility. GST and MBP fusion proteins were, respectively, purified using glutathione sepharose (GE Healthcare, UK) or amylose resin beads (New England Biolabs; UK), in batch format, following the manufacturer's instructions. Bound GST and MBP fusion proteins were eluted for 1 h (GST: 50 mM TrisHCl, 40 mM glutathione, pH 8; MBP: as suggested by manufacturer), in a volume double that of the beads. Beads were eluted three times and fusion protein-containing eluates analysed by sodium dodecyl sulphate–polyacrylamide gel electrophoresis (SDS–PAGE) using 10% Mini-Protean TGX gels (BioRad, UK) and silver staining (Pierce, Life Technologies, UK).

### 2.4 SDS–PAGE and Western blotting

Protein concentration was determined using the QuickStart Bradford Assay (BioRad, UK), following the manufacturer's protocol. Recombinant proteins were transferred to polyvinylidene difluoride (PVDF) membrane using the TransBlot Turbo system (Biorad, UK) and analysed by Western blot with anti-GST (Santa Cruz Biotech, USA) or anti-MBP antibody (New England Biolabs, UK) at 1:10,000 dilutions. Bound antibodies were detected using appropriate horseradish peroxidase (HRP)-conjugated secondary antibodies at 1:10,000 dilutions. To assess if the cloned antigens were immunogenic following natural *Campylobacter* infection, pooled serum from *Campylobacter*-infected non-vaccinated White Leghorn birds collected three weeks post-infection was used at a 1:100 dilution. Bound serum IgY was detected with an HRP-conjugated rabbit-anti chicken IgY (Sigma-Aldrich, UK) at a 1:3000 dilution. In order to analyse the subcellular localisation of SodB a Western blot of subcellular fractions (Section 2.9) was probed with sera from GST–SodB vaccinated birds as above. Blots were developed using Clarity ECL (BioRad, UK) and autoradiography (Amersham Hyperfilm ECL, GE Lifesciences, UK).

### 2.5 Vaccination and challenge experiments

All procedures were conducted under Home Office project licence PPL 60/4420, according to the requirements of the Animal (Scientific Procedures) Act 1986, with the approval of local ethical review committees. A total of 180 White Leghorn chickens, obtained on the day of hatch from a Home Office licensed breeding establishment were used. Eggs were incubated and hatched under specified-pathogen free conditions. Animals were housed in groups of up to 20 in colony cages. Groups were of mixed sex and were wing-tagged for individual identification. Water and sterile irradiated feed based on vegetable protein (DBM Ltd., UK) was provided *ad libitum*.

Three separate trials were conducted, each including vaccination with GST or GST–CjaA as negative and positive controls respectively, alongside GST–SodB. Data for the GST and GST–CjaA control groups was available from an additional experiment that did not test GST–SodB concomitantly but which had an identical design to the three experiments that tested GST–SodB in parallel. The experimental design was essentially as described [12], but with the following modifications. White Leghorn chickens rather than Light Sussex birds were used and GST–CjaA was used rather than a 6xHis–CjaA fusion [12]. Briefly, twenty birds were used per experiment per group. A mechanical dispenser and high accuracy syringes (Hamilton-Bonaduz, Switzerland) were used for both vaccinations and oral gavage. Vaccination was subcutaneous in volumes of 50 μl on each side of the thorax, using 1″, 21 gauge needles. Antigen preparations were mixed in a 1:1 ratio with TiterMax Gold^®^ (Sigma-Aldrich, UK) and each bird received 4.3 × 10^−10^ mole of recombinant protein for parity with our earlier studies using 6xHis–CjaA [12]. Birds were given the primary vaccination on the day of hatch, an identical booster 14 days later and challenged with 10^7^ colony-forming units (CFU) of *C. jejuni* M1 at 28 days post-hatch (dph) by oral gavage. Starting one week after challenge, between 4 and 6 birds were removed at weekly intervals to enumerate caecal *Campylobacter* by plating 100 μl of 10-fold serial dilutions of caecal contents in phosphate-buffered saline (PBS) on mCCDA plates. At the same time, samples of blood and bile were collected for the measurement of humoral responses. Blood was stored at −4 °C overnight to allow coagulation, after which blood cells were pelleted by centrifugation at 3000 × *g* for 10 min. Serum was collected and stored at −80 °C until use.

### 2.6 Analysis of humoral immune responses following vaccination

Enzyme-linked immunosorbent assays (ELISAs) were carried out to measure antigen-specific serum IgY and secretory bile IgA (sIgA) against SodB and CjaA. In order to improve the specificity of detection of antigen-specific antibodies, MBP fusions were used as the capture antigen in these assays. The assays were done as previously described [12], however no blocking step was used for the measurement of serum IgY. Coating conditions were optimised using chequerboard analyses for IgY and IgA individually. To analyse serum IgY, 96 well plates were coated with 0.5 μg/ml of MBP–CjaA or 2 μg/ml MBP–SodB. Serum was diluted at 1:500 for GST–CjaA and 1:250 for GST–SodB vaccinated birds. To analyse secretory IgA (sIgA), each recombinant protein was coated at a concentration of 1 μg/ml and a 1:250 bile dilution was used.

### 2.7 Immunofluorescence microscopy

For assessment of subcellular localisation of SodB within *C. jejuni* 11168H cells pooled serum from SodB-vaccinated chickens collected before challenge was used at a 1:500 dilution to stain *Campylobacter* cells bound to poly-l-lysine treated glass cover slips. Where indicated, bacterial cells were permeabilised with 10% (v/v) Triton X-100 for 30 min. A goat anti-chicken IgY conjugated to AlexaFluor-488 secondary antibody (Abcam, UK) was used for detection at a 1:500 dilution. Cover slips were mounted on glass slides using Prolong Gold (Life Technologies). Images were captured using fluorescent and light microscopy on a Leica DML (Leica, Germany) microscope.

### 2.8 Generation of subcellular fractions of *C. jejuni*

For the preparation of the periplasmic fraction of *C. jejuni*, an osmotic shock procedure was used as described [17], with modifications given in Supplementary Material. The outer membrane and inner membrane preparations were made as described [18] with modifications as given in Supplementary Material. Subcellular fractions of *C. jejuni* 11168H were used as preparations of high purity were already available from other work.

### 2.9 Statistical analysis

Statistical analyses were performed using Minitab 17 (Minitab, UK). Individual caecal *Campylobacter* counts were logarithmically transformed and the arithmetic mean was calculated. Significant reductions compared to control groups were determined using *post-hoc* Dunnet tests following fitting of a second order hierarchical general linear model (GLM) that took into account interactions between time of sample collection and treatment group. The first two outliers in each group, as identified by the GLM as having high residuals of over 2.5 log_10_ CFU/g, were removed from the data. To test whether humoral immune responses were significantly induced relative to control birds the mean OD_450_ reading was calculated and a two-tailed Student's *T*-test used to detect significant increases in antibody levels. Antigen-specific fold changes in OD_450_ of serum IgY in individual birds were calculated by dividing the OD_450_ measures in each vaccinated bird by the average of the control group calculated at each sampling time-point. Correlations between serum IgY levels and caecal *Campylobacter* counts in individual birds were assessed by fitting of a linear regression to the data. *P* values of ≤0.05 were considered significant.

## 3 Results

### 3.1 Recombinant protein purification and validation

**Fig. 1:**
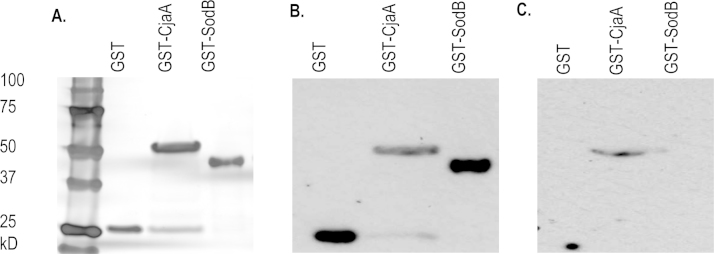
*Purity and immunogenicity of vaccine preparations*. (A) SDS–PAGE analysis of GST, GST–CjaA and GST–SodB vaccine preparations, followed by silver staining. (B) Western blot of the vaccine preparations in panel A using anti-GST antibody. (C) Western blot of the vaccine preparations in panel A using sera from *Campylobacter*-infected but non-vaccinated chickens. For all the lanes presented a total of 1 μg protein was used, in a final volume of 20 μl, mixed 1:1 with denaturing running buffer.

The preparations of GST and GST–SodB used in all vaccination trials and a typical preparation of GST–CjaA (from the first vaccination trial) are shown in Fig. 1A. In Western blots using GST-specific antibody the GST–SodB preparation was detected as a single species whereas the GST–CjaA preparation appeared to contain a species of the size of GST only as well as the dominant GST–CjaA fusion protein (Fig. 1B). A similar pattern was observed previously when CjaA was expressed as a fusion to TetC [12]. MBP fusions were similarly validated with an anti-MBP antibody (data not shown). To determine if the proteins are recognised during *C. jejuni* infection, pooled serum from unvaccinated chickens challenged with *C. jejuni* M1 was used for Western blots. The serum reacted to GST–CjaA but not GST–SodB or GST alone (Fig. 1C), implying that of the antigens tested only CjaA is naturally immunogenic following *Campylobacter* infection with the M1 strain, at least at the limit of detection of the method. However, owing to use of denaturing SDS–PAGE only linear epitopes would be detected.

### 3.2 Vaccination of chickens with recombinant SodB elicits a statistically significant reduction in caecal *Campylobacter* colonisation

**Fig. 2:**
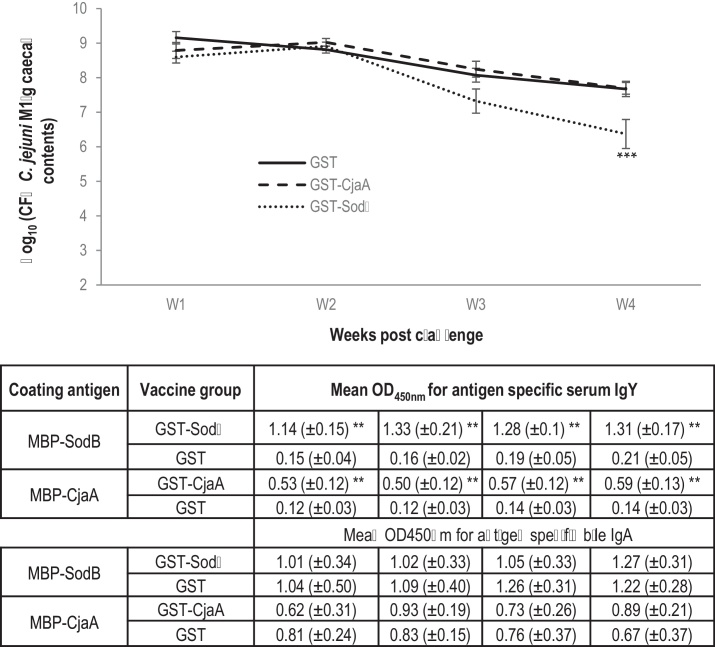
*Purified recombinant SodB-based vaccine, but not a CjaA-based vaccine, was protective against homologousCampylobacterchallenge in chickens.* Data represent the arithmetic mean of log transformed caecal counts of *C. jejuni* from a minimum of 3 independent trials where each antigen was tested concomitantly, each using 3–6 birds per time interval, ± the standard error of the mean (SEM). Data for the GST and GST–CjaA control groups was available from an additional experiment that did not test GST–SodB concomitantly but which had an identical design to the three experiments that tested GST–SodB in parallel. Samples were collected at weekly intervals at post-mortem examination and as a result the lines are inferred to reflect the course of excretion, and are not longitudinal values from the same animals. Values in the table are mean CjaA- and SodB-specific serum IgY and bile secretory IgA levels in vaccinated and control groups measured by ELISA at the time intervals on the *x*-axis. Changes compared to the GST control group were determined using a generalised linear model (*R*^2^ = 0.47) and *post-hoc* Dunnet's tests. Statistically significant differences to the GST control group are denoted with and *** for *P* ≤ 0.001.

We evaluated the impact of vaccination of chickens with GST–SodB on protection against homologous challenge with *C. jejuni* in three independent trials. In these trials, GST–CjaA was tested concomitantly as CjaA was previously demonstrated to be protective when given as a 6xHis-tagged recombinant protein [12]. GST alone was given as a negative control. No adverse effects of vaccination were noted in any of the experimental animals and no obvious clinical signs were induced by the challenge with *C. jejuni* M1 in any of the birds. Caecal *Campylobacter* loads were determined at post-mortem examination at weekly intervals following challenge as longitudinal sampling by cloacal swabbing is less reliable for obtaining viable counts [19]. Across 3 biological replicates (4–6 birds sampled at each time point per group, per replicate) the groups vaccinated with GST–SodB had a significantly different course of caecal *Campylobacter* colonisation compared to both the GST (*P* = 0.001) and GST–CjaA (*P* < 0.001) vaccinated groups tested across 4 biological replicates (Fig. 2A) *Post-hoc* Dunnet's tests indicated that the 1.3 log_10_ reduction observed at 56 dph in the GST–SodB vaccinated group compared to the GST vaccinated group was significant (*P* < 0.001). At 49 dph a reduction of 0.75 log_10_ was also observed compared to the GST group, however, this was not statistically significant (*P *= 0.19). Significant reduction were observed in the GST–SodB vaccinated group compared to the GST–CjaA vaccinated group at both 49 (*P* = 0.048) and 56 dph (*P *< 0.001). No reductions were observed in the GST–CjaA vaccinated birds relative to those given GST alone at any time interval.

### 3.3 Both SodB- and CjaA-based vaccines induced antigen-specific antibody responses following vaccination

**Fig. 3:**
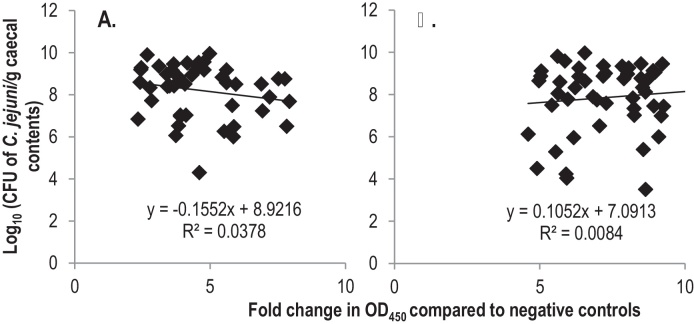
*Serum IgY levels and caecal Campylobacter counts do not correlate in individual birds*. The data represent a linear regression in individual birds of caecal *Campylobacter* counts on fold changes in OD_450 nm_ values in ELISAs measuring antigen-specific serum IgY levels in the GST–CjaA (panel A) and GST–SodB (panel B) vaccinated birds. Fold changes in serum IgY (indicated on the *x* axis) were calculated by dividing the OD_450 nm_ reading for individual birds in the *Campylobacter* vaccine groups by the mean reactivity of the GST only vaccinated group to the corresponding antigen.

In order to measure antigen-specific humoral responses, serum IgY and bile IgA responses against *C. jejuni* M1 antigens in vaccinated birds were quantified by ELISA. *Campylobacter* antigens were expressed as MBP fusions to separate responses to *C. jejuni* antigens from the GST fusion partner. A significantly higher level of antigen-specific serum IgY was induced in the GST–SodB and GST–CjaA vaccinated groups compared to GST vaccinated groups at all time-points measured (Fig. 2). However, no significant induction of antigen-specific bile IgA was detected in either of the vaccinated groups at any of the time points (Fig. 2), similar to previous observations [12]. Further, at the level of individual birds the magnitude of antigen-specific serum IgY responses did not correlate with caecal *Campylobacter* counts for either of the antigens (Fig. 3).

### 3.4 SodB is an intracellular protein in *C. jejuni* cells

**Fig. 4:**
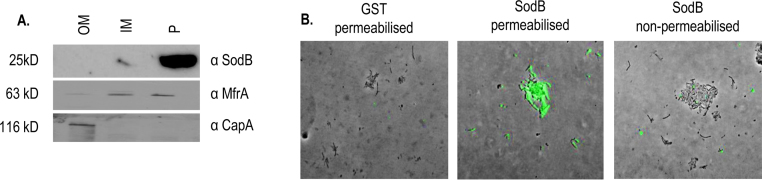
*SodB is absent from the outer membrane and surface of C. jejuni*. (A) Immunoblotting of inner membrane, outer membrane and periplasmic subcellular fractions of *C. jejuni* 11168H with sera from GST–SodB vaccinated birds collected immediately prior to challenge. (B) Immunofluorescence microscopy of *C. jejuni* 11168H stained with sera from the same birds used in panel A and from GST only vaccinated birds as a negative control.

The subcellular localisation of SodB within *C. jejuni* 11168H cells was determined by Western blotting of subcellular fractions and immunofluorescence microscopy in order to assess the likelihood of directly neutralising antibodies playing a role in protection following vaccination. Sera from GST–SodB vaccinated birds detected SodB only in the periplasmic fraction of *C. jejuni* 11168H but not within the outer or inner membrane fractions (Fig. 4A). The purity of the fractions was demonstrated by Western blotting with αCapA (an outer membrane auto-transported adhesin, [20]) or αMfrA (a periplasmic fumarate reductase subunit associated with an inner membrane complex, [21]; Fig. 4A). As a cytoplasmic fraction was not examined we cannot be certain that SodB exists only in the periplasm of *C. jejuni*. Immunofluorescence microscopy using serum from GST–SodB vaccinated birds detected specific staining of permeabilised *C. jejuni* cells, but not non-permeabilised cells (Fig. 4B), supporting a lack of surface exposure of SodB, at least within the limit of detection of the method. Secretion of SodB was not assessed in this study. SodB lacks a predicted signal peptide for secretion in *Campylobacter* and no evidence of other iron-base superoxide dismutases being secreted in bacteria is available in the literature.

## 4 Discussion

Towards the aim of developing a vaccine to control *Campylobacter* in its primary reservoir, we evaluated the efficacy of purified GST–SodB in reducing *Campylobacter* colonisation in chickens. The vaccine reduced caecal colonisation by approximately 1 log_10_ at 49 and 56 dph relative to GST-vaccinated birds, however only the reduction at 56 dph proved to be statistically significant. The GST–CjaA vaccine was not protective, unlike our previous study [12], however this could be due to changes in the design of the experiments. For example the line of birds used was White Leghorn rather than Light Sussex and the fusion partner and affinity purification processes were different. Further, the existence of a truncation of GST–CjaA (Fig. 1A and B) meant that the molar quantity of CjaA received by the GST–CjaA vaccinated birds was lower than when the 6xHis–CjaA fusion was evaluated, and the current study used the Gold version of TiterMax^®^ as adjuvant which is further optimised for the promotion of humoral responses. The lack of a protective effect using GST–CjaA should not cast doubt on the use of GST fusion vaccines for control of *Campylobacter*. Previous studies have demonstrated protection against colonisation and clinical symptoms using GST–PorA in a murine model of campylobacteriosis [22] and protection against colonisation through the use of a combined GST and 6xHis-tagged FlpA vaccine in a chicken model [13].

Though the GST–SodB-based vaccine was protective, both immunofluorescence microscopy of whole *C. jejuni* cells and Western blotting of subcellular fractions indicated the absence of SodB from the bacterial surface. This is consistent with the subcellular localisation of SodB within *E. coli* [23], and indicates that directly neutralising antibodies binding to the bacteria may not play a major role in protection. This is supported by bacteria not being agglutinated when mixed 1:1 (v/v) with serum from GST–SodB vaccinated birds collected at the time of challenge (data not shown). Furthermore, despite the significant induction of humoral responses by the SodB- and CjaA-based vaccines compared to GST-vaccinated birds, levels of antigen-specific serum IgY levels did not correlate with caecal *Campylobacter* counts in individual birds (Fig. 3), nor was the peak of antigen-specific IgY coincident with the timing of the protective effect (Fig. 2). In addition, both vaccines failed to induce antigen-specific detectable biliary IgA at any of the time intervals studied. Further characterisation of the nature and consequences of cell-mediated and humoral responses in protection against *Campylobacter* colonisation will help to refine vaccine design of vaccines and inform the selection of adjuvants. Vaccination of transgenic chickens lacking the Ig heavy chain J segment [24] would allow the role of antibody in vaccine-mediates protection to be formally established.

Quantitative risk assessments predict that even a relatively modest hundred-fold reduction of *Campylobacter* on broiler carcasses could reduce the incidence of human disease due to chicken consumption by 30-fold [25]. Even though protective vaccines against *Campylobacter* in chickens have been described, they each present drawbacks that hinder field application. Some are costly to produce [26], others pose the challenge of attenuated live vectors that persist at the point of entry into the food chain [11], [12], and others require very high doses to obtain protection [13]. The GST–SodB vaccine described herein has the advantages of high sequence conservation and high solubility in aqueous medium, making it easy to produce and deliver under experimental conditions. However, a successful field vaccine is likely to require vectoring due to benefits in cost and ease of use. Our study shows proof-of-potential for anti-*Campylobacter* vaccination using SodB in chickens and adds an additional protective antigen to the limited repertoire of those described in the literature to date.

## Conflict of interest statement

None of the authors have any conflicts of interest. Zoetis did not participate directly in the design and evaluation of the vaccines described.
