# Supplementary material for: Superoxide dismutase SodB is a protective antigen against Campylobacter jejuni colonisation in chickens
Source: Vaccine. 2015 Nov 17;33(46):6206–11. doi: 10.1016/j.vaccine.2015.09.100 (PMC4654421; doi:10.1016/j.vaccine.2015.09.100)
Supplement: Supplementary file 1 [file mmc1.docx]

**Materials and Methods: Generation of subcellular fractions**

*Periplasmic fraction*

For the preparation of the periplasmic fraction of *C. jejuni*, an osmotic shock procedure was used. *C. jejuni* 11168H cultures in early stationary phase were harvested by centrifugation (8,000g, 10 min, 4 °C) and resuspended in 20 ml buffer containing 20% (w/v) sucrose, 30 mM Tris-HCl pH8, 1mM EDTA and incubated with gentle shaking for 30 min. The cells were pelleted by centrifugation at 10,000g, for 10 min at room temperature and then re-suspended in 10 ml ice-cold 10 mM Tris-HCl pH8. Following incubation with gentle shaking (20 rpm, 2 hr, 4°C), cells were pelleted by centrifugation at 15,000g for 25 min at 4 °C, and the supernatant containing the periplasmic proteins was carefully removed and stored at -20 °C.

*Membrane fractions*

To prepare the inner and outer membrane fractions, *C. jejuni* 11168H cells were cultured to early stationary phase and harvested by centrifugation (15,000g, 20 min, 4 °C) then re-suspended in 10 ml 10 mM HEPES pH 7.4. The cells were disrupted by sonication with 6 x 15 sec pulses at an amplitude of 16 microns using a Soniprep 150 ultrasonic disintegrator (MSE, UK) and then pelleted by centrifugation (15,000g, 20 min, 4°C) to remove unbroken cells and debris. In order to isolate the outer and inner membrane, total membranes were first pelleted by ultracentrifugation (100,000g, 1 hr, 4 °C) in a Ti70.1 rotor (Beckman Coulter, UK) and the pellet washed twice with 5 ml of 10 mM HEPES pH 7.4. The membrane pellet was re-suspended in 1 ml 10 mM HEPES pH 7.4. To solubilise the inner membrane, an equal volume of 2% (v/v) sodium N-Lauryl sarcosinate dissolved in 10 mM HEPES buffer was added with gentle mixing. Following incubation at 37 °C for 30 min, the mixture was pelleted by centrifugation (15,000g, 30 min, 4 °C). The supernatant (solubilized inner membrane) was collected and stored at -20 °C and the outer membrane pellets were resuspended in 10 mM HEPES buffer pH 7.4, washed and re-pelleted twice. Finally, the pellet was resuspended and dispersed in 0.5-1 ml of 10 mM HEPES buffer pH 7.4 and stored at -20 °C.
